# Effects of Silicon-Limitation on Growth and Morphology of *Triparma laevis* NIES-2565 (Parmales, Heterokontophyta)

**DOI:** 10.1371/journal.pone.0103289

**Published:** 2014-07-23

**Authors:** Kazumasa Yamada, Shinya Yoshikawa, Mutsuo Ichinomiya, Akira Kuwata, Mitsunobu Kamiya, Kaori Ohki

**Affiliations:** 1 Faculty of Marine Bioscience, Fukui Prefectural University, Obama, Japan; 2 Faculty of Environmental & Symbiotic Sciences, Prefectural University of Kumamoto, Tsukide, Higashi, Kumamoto, Japan; 3 Tohoku National Fisheries Research Institute, Shinhama-cho, Shiogama, Japan; Laval University, Canada

## Abstract

The order Parmales (Heterokontophyta) is a group of small-sized unicellular marine phytoplankton, which is distributed widely from tropical to polar waters. The cells of Parmales are surrounded by a distinctive cell wall, which consists of several siliceous plates fitting edge to edge. Phylogenetic and morphological analyses suggest that Parmales is one of the key organisms for elucidating the evolutionary origin of Bacillariophyceae (diatoms), the most successful heterokontophyta. The effects of silicon-limitation on growth and morphogenesis of plates were studied using a strain of *Triparma laevis* NIES-2565, which was cultured for the first time in artificial sea water. The cells of *T. laevis* were surrounded by eight plates when grown with sufficient silicon. However, plate formation became incomplete when cells were cultured in a medium containing low silicate (ca. <10 µM). Cells finally lost almost all plates in a medium containing silicate concentrations lower than ca. 1 µM. However, silicon-limitation did not affect growth rate; cells continued to divide without changing their growth rate, even after all plates were lost. Loss of plates was reversible; when cells without plates were transferred to a medium containing sufficient silicate, regeneration of shield and ventral plates was followed by the formation of girdle and triradiate plates. The results indicate that the response to silicon-limitation of *T. laevis* is different from that of diatoms, where cell division becomes inhibited under such conditions.

## Introduction

The order Parmales (Heterokontophyta) is a group of small-sized (2–5 µm in diameter) unicellular marine phytoplankton, which is distributed widely from tropical to polar waters [1], [2]. The cells of Parmales are surrounded by 5 or 8 siliceous plates that fit edge to edge. Earlier morphological studies using a scanning electron microscope (SEM) suggested that they were resting cysts of siliceous loricate choanoflagellates [3]. Later, they were found to be photosynthetically active vegetative cells and not cysts, because they had a large chloroplast and only a small amount of storage material [4]. Based on the morphological properties observed with natural samples, Booth and Marchant (1987) established a new order Parmales containing three genera, *Pentalamina, Tetraparma* and *Triparma*, within the class Chrysophyceae [1]. Recently, the first culture of Parmales was successfully established by Ichinomiya and his coworkers (*Triparma laevis* NIES-2565) [5]. Molecular phylogenetic analysis of SSU rDNA and *rbc*L genes revealed that *T. laevis* was situated in the Bolidophyceae clade, and not in Chrysophyceae. Bolidophycean algae are small, naked flagellates that have been recognized as the closest sister group of diatoms (Bacillariophyceae) [6], [7].

In heterokontophyta, structured siliceous cell walls occur in diatoms, Synurophyceae (separated from Chrysophyceae) [8] and Dictyochophyceae as well as Parmales. The siliceous cell wall of Synurophyceae is made up of scales that are arranged loosely outside of the plasma membrane, while in Dictyochophyceae the cell wall consists of a skeleton that is made up of hollow, tubular rods fused together to form a network. In diatoms, scale-covered cells appear only at the auxospore stage. The vegetative cells of diatom are characterized by a siliceous cell wall called a frustule, which consists of two box-like valves, each accompanied by a series of girdle bands. A skeletal structure is not found in diatoms. As frustule-like cell walls are not found in other Heterokontophyta, the evolutional origin of diatoms and their distinctive silica frustule has long been puzzling. Round and Crawford (1981) proposed the hypothesis that the frustule arose from silica-scaled algae, where apical siliceous scales increased in size during the course of evolution to form valves at either ends of the cell, with the rest of the scales being modified to form girdle bands [9]. In 1989, after a new order Parmales had been established, Mann and Marchant compared the siliceous wall structure of Parmales and diatoms, and pointed out their morphological similarities, e.g. between the round or shield plates of Parmales and the valves of centric diatoms, the medial rib and keel of the girdle plates of *Triparma* and pars media of diatom girdle bands, and the plate patterning in *Triparma columacea* and *T. retinervis* and that of the auxospore scales of some *Melosira* species [10].

Diatoms require silicon for cell division because of the unique structure and morphogenesis of their frustule [11], [12]. It has been shown that their cell cycle is interrupted at the G-stage under silicon-limitation [13], [14], [15]. In other words, siliceous cell wall formation and the cell cycle are tightly coupled in most diatoms. In contrast to diatoms, the effects of silicon-limitation appear to be on cell wall morphogenesis rather than growth in most other siliceous-walled algae. For example, *Synura petersenii* (Synurophyceae) was able to grow without forming siliceous scales under silicon-limitation [16]. Cells without siliceous skeletons were observed under silicon-limited environments in *Dictyocha speculum* (Dictyochophyceae), though it was not clear whether they were actively growing or not [17].

Because Parmales are the most closely related organisms to diatoms among Heterokontophyta having siliceous cell wall, physiological studies in response to silicon-limitation with reference to siliceous cell wall morphogenesis of Parmales can provide a new insight into the evolution of siliceous-walled algae in Heterokontophyta.

The isolated strain of *T. laevis* used in this study was maintained in enriched sea water (f/2 medium [18]), which is a common approach used for culturing and physiological studies of marine phytoplankton. However, it is impossible to obtain accurate and reproducible concentrations of salts and nutrients using enriched sea water media. Therefore, our first aim was to establish cultures of *T. laevis* in an artificial sea water medium that showed the same growth rate and yield as in enriched sea water medium. Once this was achieved, our aim was to characterize the growth and siliceous cell wall morphogenesis of *T. laevis* in response to silicon-limitation.

## Materials and Methods

### Materials

*Triparma laevis* NIES-2565 was the same strain used in a previous study [5]. After isolation, the culture had been maintained in f/2 medium [18] under 12 h light (using daylight type fluorescent lamps, 30 µmol m^−2 ^s^−1^) and 12 h dark cycles at 5°C.

### Establishment of culture in artificial sea water medium

Five types of artificial sea water media were tried; (1) ASP_7_ [19], (2) ASP_7_ but with the concentration of tris hydroxymethyl aminomethane HCl (Tris, pH 8.0) reduced to one-tenth (0.1 g·L^−1^), (3) modified Aquil medium [20], (4) same as (3) but with Tris added at the same concentration as ASP_7_ (1 g·L^−1^) and (5) same as (4) but with the Tris concentration reduced to one-tenth. For the first inoculum cells were maintained in f/2 medium. About 0.5 ml of cell suspension was inoculated into test tubes containing about 15 ml of artificial sea water medium, and incubated under the same light and temperature conditions used for maintenance of cultures in f/2 medium. The cells were cultured for at least 100 generations in the same medium before the experiment, then they were transferred to fresh medium of the same type when still in the exponential growth phase.

To estimate cell densities, an aliquot of cell suspensions was withdrawn aseptically from each culture every day, fixed with glutaraldehyde (1% v/v, final concentration) and stored at 4°C until analysis. A certain amount of fixed cells (<1 ml) were collected onto a polycarbonate membrane filter (0.2 µm pore size; Advantec K0220A047; Toyo Roshi Kaisha, Tokyo, Japan), and the number of autofluorescent cells was directly counted under an epifluorescent microscope (BX51 with filter-set; BP460–495/BA510IF/DM505, Olympus Co., Tokyo, Japan). To determine the cells on the filtration area, number of all cells in 10 randomly selected fields were counted. Results were the mean ± standard deviation (SD) of triplicate cultures.

### Experimental cultures under silicon-limitation

Media with various concentrations of silicate (added as Na_2_SiO_3_·9H_2_0, Nacalai Tesque Inc. Kyoto, Japan; 1 µM, 10 µM and 100 µM, which we refer to as 1–100 µM-silicate medium etc. here after) were used. The experiments were based on a batch culture system, so silicate concentrations in the media were reduced as cell abundance increased. Therefore, the values indicated are the initial concentrations of silicate, unless otherwise stated. Cells in the exponential growth phase were inoculated at a cell density of ca. 10^4^ cells ml^−1^ into fresh medium, and then cultured under the same conditions as those used for the maintenance cultures. Cells were pre-cultured over at least five generations in the same silicate concentration as those used for the experiments. Polycarbonate culture vessels with low concentrations of silicate (1 and 10 µM) were used to avoid any contamination of silicon from glassware. The specific growth rate, µ (d^−1^), was determined from the increase of cell densities during the exponential growth phase. The data were the mean ± SD from two experiments with triplicate cultures.

### Estimation of silicate concentration in the medium and cell silicon quota

To determine the actual silicate concentrations in the medium, several ml of cell suspension were withdrawn from each culture aseptically at the time indicated, then kept at −40°C after the cells were removed by filtration. To estimate the cell silicon quota, cells in the exponential phase were collected onto a polycarbonate filter and solubilized in 0.2 M NaOH at 100°C for 15 minutes, and then neutralized by addition of 0.2 M HCl. Silicon concentrations were measured spectrophotometrically at 810 nm using a silicomolybdate method [21]. Concentrations below 0.7 µM could not be estimated correctly by our measurement systems. The data were the mean ± SD from triplicate cultures.

### Morphological observations under SEM

Cells were fixed and collected by the same methods used for determination of cell densities. Cells on a filter were then dehydrated through a graded ethanol series, substituted with *t*-butyl alcohol, and finally freeze-dried (JFD-300, JEOL, Tokyo, Japan [22]). The dried cells coated with gold-palladium using an ion sputter (JFC-1500, JEOL). Samples were observed under SEM (SU1510, Hitachi High-Technologies Co., Tokyo, Japan).

For the quantitative determination of morphological changes under silicon-limitation, a hundred randomly selected cells from triplicate cultures were categorized into 4 types (see Results) by their SEM images, and then the mean percentage of each type was calculated. The data was analyzed by t-test.

### Quantitative and qualitative analyzes of cell wall regenerated cells

Cells grown in 1 µM-silicate medium and lost siliceous cell wall were inoculated into 100 µM-silicate medium containing 2-(4-pyridyl)-5-[(4-(2-dimethylaminoethylaminocarbamoyl)methoxy)-phenyl] oxazole (PDMPO, final concentration 0.125 µM [5]). PDMPO is incorporated into diatom cells and co-deposited with silicon into the solid silica matrix of the newly produced silica plates. It produces an intense yellowish-green fluorescence under ultra violet (UV) excitation wherever silicic acid is polymerized forming biogenic silica [23]. Cell suspensions were harvested every day and fixed by the same methods used for estimation of cell densities. PDMPO fluorescence excited by UV was observed under the same epifluorescent microscope used for cell counts, but with a different filter set (BP330–385/BA420/DM400). More than 200 cells were counted for quantitative estimation of PDMPO-fluorescent cells during regeneration of the cell wall. Data are the mean ± SD from triplicate cultures. Morphological changes during cell wall regeneration were observed using a SEM.

## Results

### The growth in artificial sea water media

**Figure 1:**
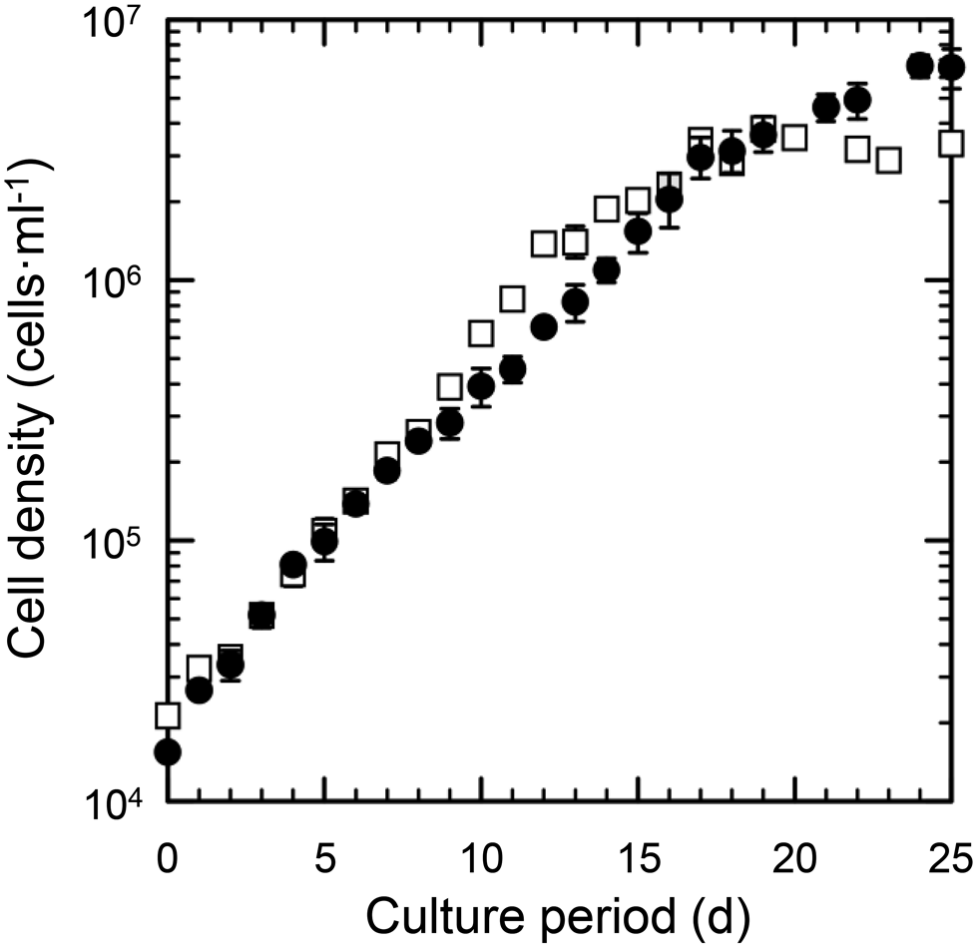
Growth of *Triparma laevis* NIES-2565 in batch culture. Open squares and closed circles are for enriched sea water medium and artificial sea water medium, respectively. Results are the mean ± SD of triplicate cultures. Error bars are omitted when ± SD was too small to be shown.

Among the five artificial sea water media tried, only modified Aquil without Tris provided stable growth of *Triparma laevis* NIES-2565. The growth rate (µ), 0.28±0.014 d^−1^, was the same and the maximum cell yield, 6.7×10^6^ cell ml^−1^, was slightly higher than those of enriched sea water medium (f/2, Figure 1). Therefore, the artificial sea water medium used for all experiments was the modified Aquil without Tris.

### Effects of silicon-limitation on the growth rate and cell silicon quota

**Figure 2:**
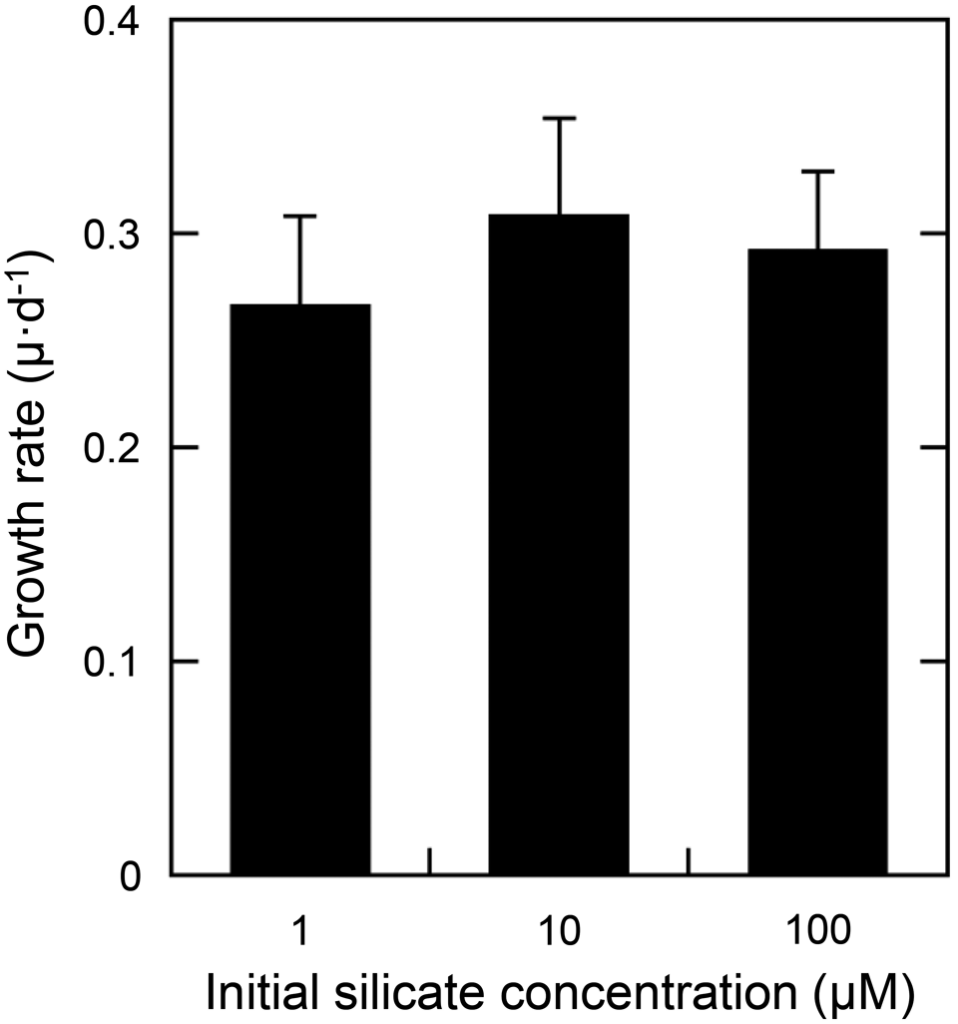
Effects of silicate concentration on the growth of *Triparma laevis* NIES-2565. Results are the mean ± SD of two experiments with triplicate cultures.

**Figure 3:**
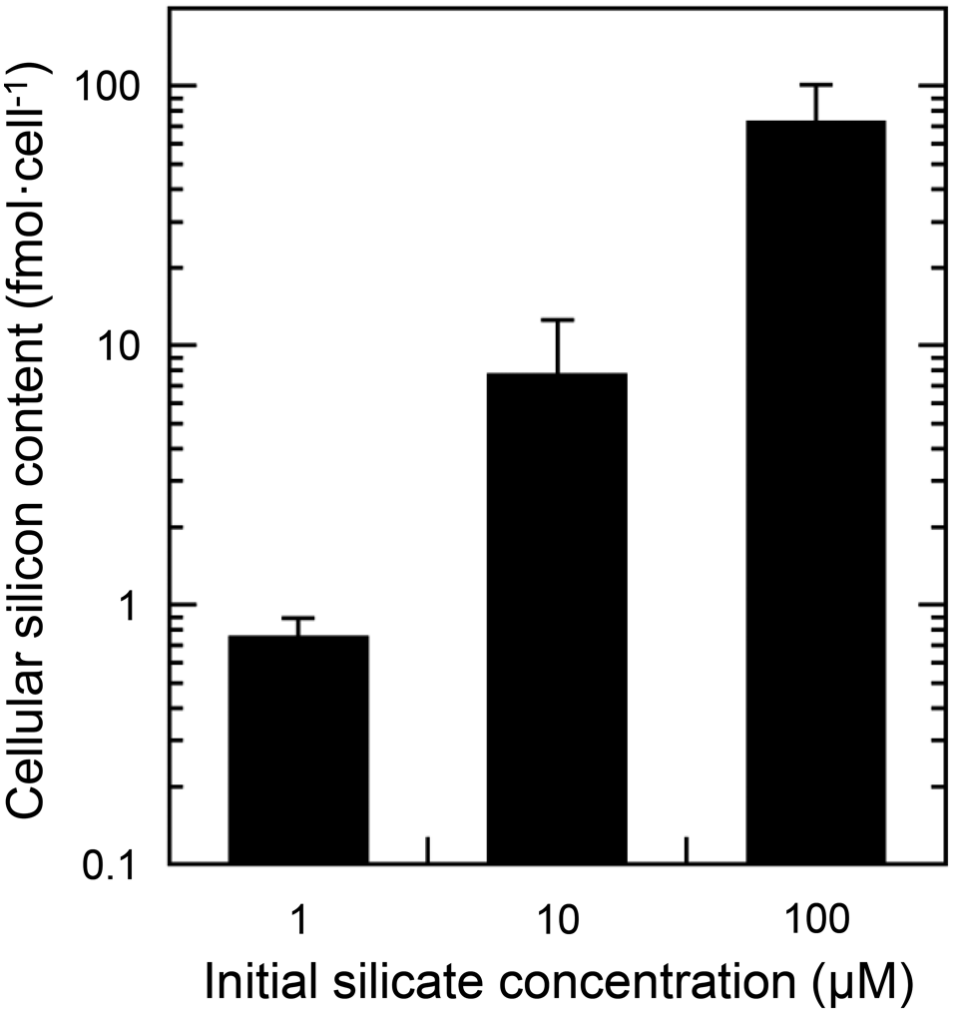
Effects of silicate concentration on cellular silicon contents of *Triparma laevis* NIES-2565. Results are the mean ± SD of triplicate cultures.

The growth rate of *T. laevis* was not affected significantly by silicate concentration between 1 to 100 µM (*n* = 6, *P*<0.05, Figure 2). However, the cell silicon quota (measured during the exponential growth phase) changed dramatically, when cells were grown in media with reduced silicate concentration (Figure 3). For example, the cell silicon quota grown in 1 µM-silicate medium (0.8±0.14 fmol cell^−1^) contained about one-hundredth of that in 100 µM-silicate medium (72.6±29.0 fmol cell^−1^).

### Morphological changes under silicon-limitation

**Figure 4:**
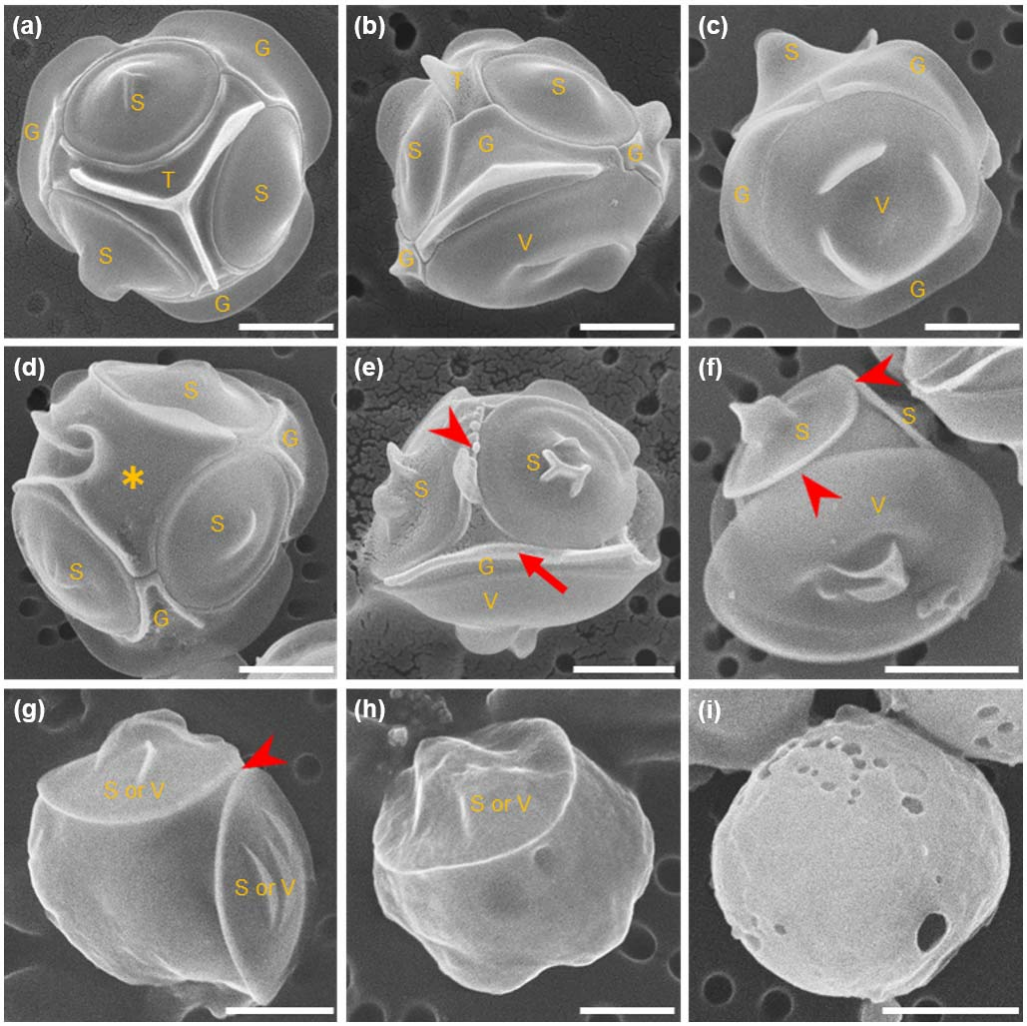
Effects of silicate concentration on morphology of siliceous cell wall of *Triparma laevis* NIES-2565. Cells cultured in 100 µM-silicate medium (a)–(d) or in 10 µM-silicate medium (e)–(i). (a)–(c) Cells viewed from different directions; cell wall is composed with three shield plates (S), one ventral plate (V), three girdle plates (G) and one triradiate plate (T). (d) Few cells have a plate, presumably triradiate plate, with abnormal shape (asterisk). (e) Cell with incomplete girdle plate (arrow) and without triradiate plate. Two shield plates seem to contact directly (arrow head) (f) Cell with ventral and shield plates but without girdle plate. Ventral plate and shield plates seem to contact directly each other (arrow head). (g) Cell with two shield and/or ventral plates. Two plates seem to contact directly (arrow head). (h) Cell with only one shield or ventral plate. (i) Cell without any plates. Bars indicate 1 µm.

**Figure 5:**
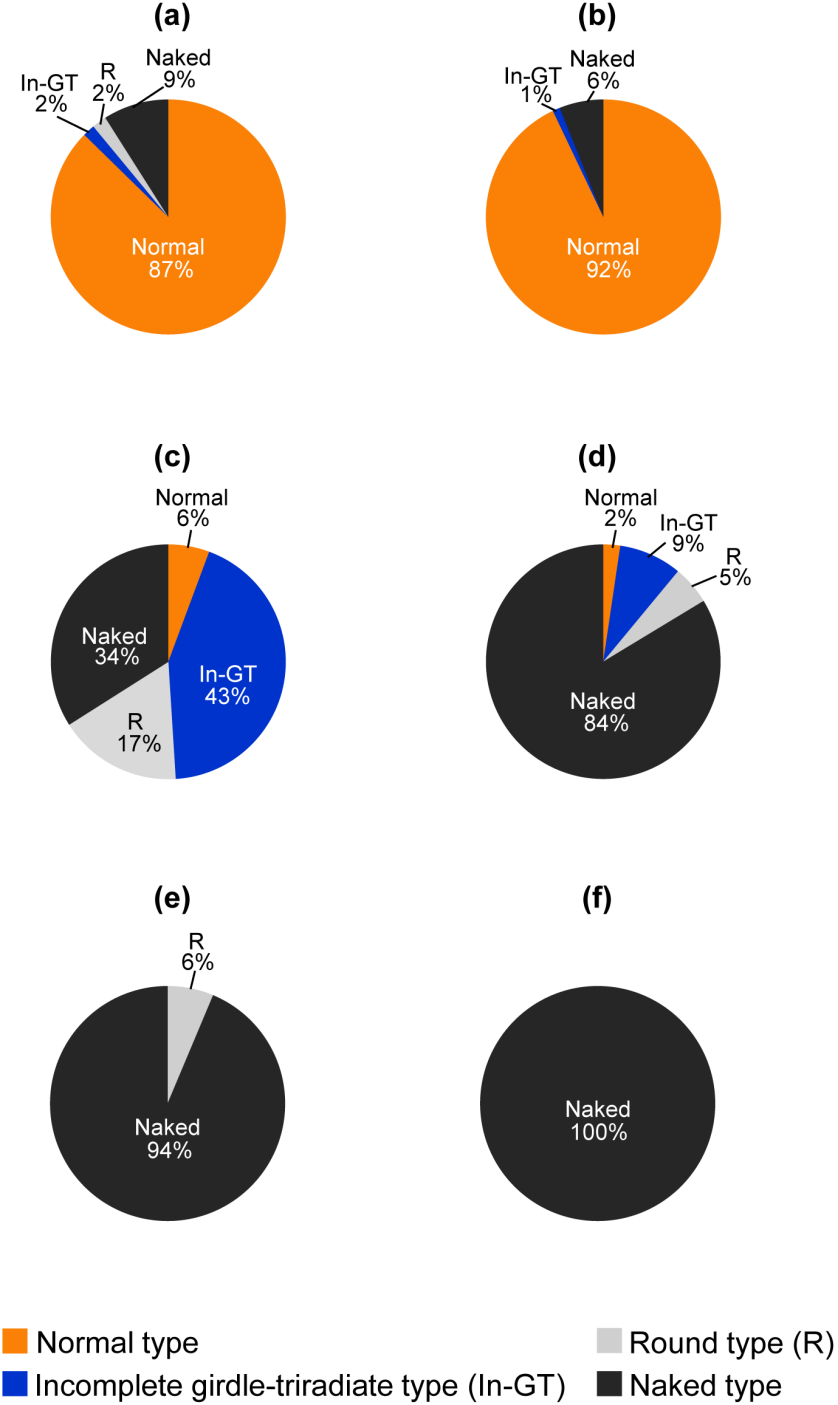
Quantitative analysis of silicate plate morphogenesis of *Triparma laevis* NIES-2565 under silicon-limitation. Cells cultured in 100, 10 or 1µM -silicate medium for 4 d (a, c and e respectively) and 12 d (b, d and f respectively). The measured silicate concentrations at the time when cells were harvested were 96.9, 89.5, 6.8, 1.1, 1.3 and lower than 0.7 µM (undetectable by silicomolybdate methods) for (a), (b), (c), (d), (e) and (f), respectively. Categorizations of each cells are as follows; Normal type: the cells with eight complete plates contact each other without any gap (see Figure 4a–c), Incomplete girdle-triradiate type (In-GT): the cells with shield and ventral plates and incomplete girdle and/or triradiate plates (see Figure 4e), Round type (R): the cells without girdle and triradiate plates (see Figure 4f–h) and Naked type: the cells without any plate (see Figure 4i). Values are the mean of randomly selected 100 cells from triplicate experiments.

The siliceous cell wall of *T. laevis* consisted of 8 plates, 3 shield plates (a round shape with equal size, indicated by the S in Figure 4), 1 triradiate plate (plate with three arms fitting in between two shield plates, indicated by the T in Figure 4), 1 ventral plate (a round shape of greater diameter than the shield plates and located on the opposite side of the sphere from the triradiate plate, indicated by the V in Figure 4) and 3 girdle plates (oblong plate juxtaposed end to end to form a ring around the ventral plate between it and the other four plates, indicated by the G in Figure 4) [1], [5]. When cells were cultured in 100 µM-silicate medium, almost all cells were covered by the typical 8 plates without a gap (Figure 4a–c, 5a, b). However, a few cells did have a plate of irregular shape (Figure 4d, asterisk). A few cells without one or all plates were observed; it is probable that in this case a plate(s) was lost mechanically during the cell collection and fixation process. When cells were cultured in 10 µM-silicate medium, the ratio of cells having incomplete plate(s) and/or lost plate(s) increased (Figure 4e–i). As it is difficult to discriminate between incomplete shield and ventral plates or girdle and triradiate plates under SEM images, morphologies observed under silicon-limitation were categorized into four types as follows; cells covered with 8 complete plates without a gap (normal type, Figure 4a–c), those covered with shield, ventral, incomplete girdle and/or triradiate plate(s) (incomplete girdle-triradiate type, Figure 4e), those covered with only shield and/or ventral plate(s) (both complete and incomplete, round type, Figure 4f–h) and those without any plate (naked type, Figure 4i). Interestingly, the cells with girdle and/or triradiate plates but without shield and/or ventral plates could not be found. The ratio of each category is presented in Figure 5. When cells were cultured in 10 µM-silicate medium, the naked type cells became 34±4.4 and 84±2.1% (mean ± SD) in early (4 d culture) and late (12 d culture) exponential growth phase, respectively (Figure 5b, c). In 12 d culture, rate of naked type cell significantly higher than 4 d culture (*n* = 3, *P*<0.01). The growth and changes of silicate concentration in the medium during 12 days of 10 µM-silicate medium showed in Figure S1. Almost all cells became naked type when cells were cultured in 1 µM-silicate medium (Figure 5e, f). Morphology of naked cell in 1 µM was the same as that in 10 µM-silicate medium (Figure 4i).

### Regeneration of siliceous cell wall with resupply of silicate

**Figure 6:**
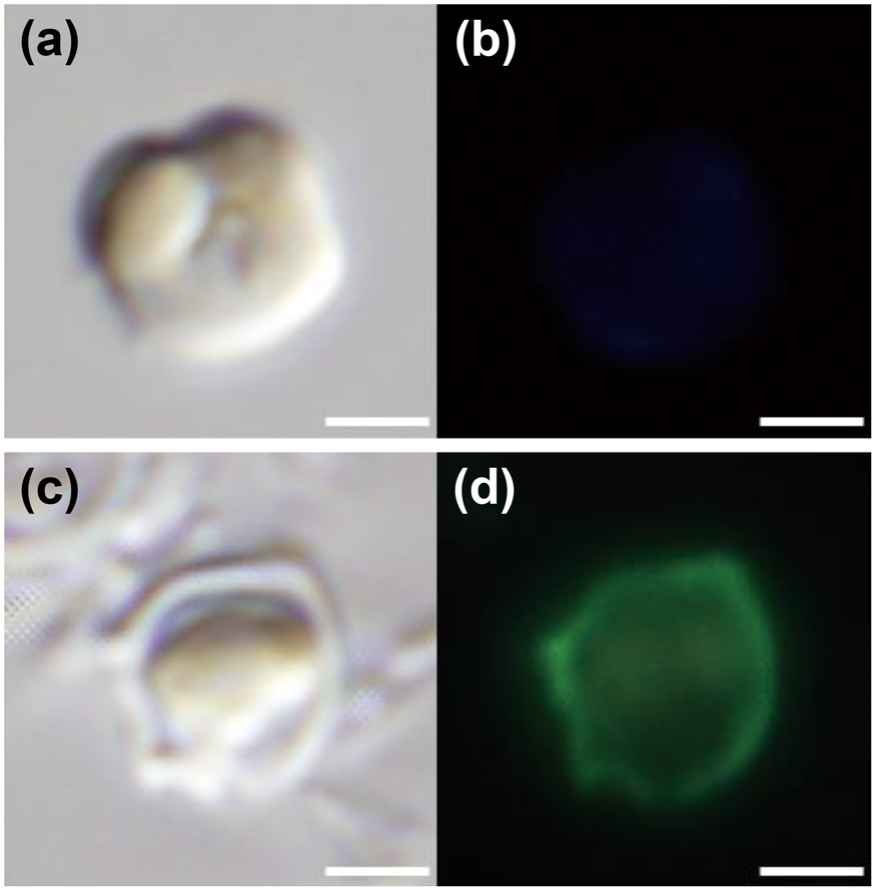
Incorporation and deposition of silicon by means of PDMPO fluorescence in *Triparma laevis* NIES-2565. (a), (b) Cells cultured in 1 µM-silicate medium. (c), (d) Cells that had lost all plates when cultured in 1 µM-silicate medium were transferred into 100 µM-silicate medium containing PDMPO and incubated for 24 h. Cells observed under light or epifluorescent microscope for (a), (c) and (b), (d), respectively. Bars indicate 1 µm.

**Figure 7:**
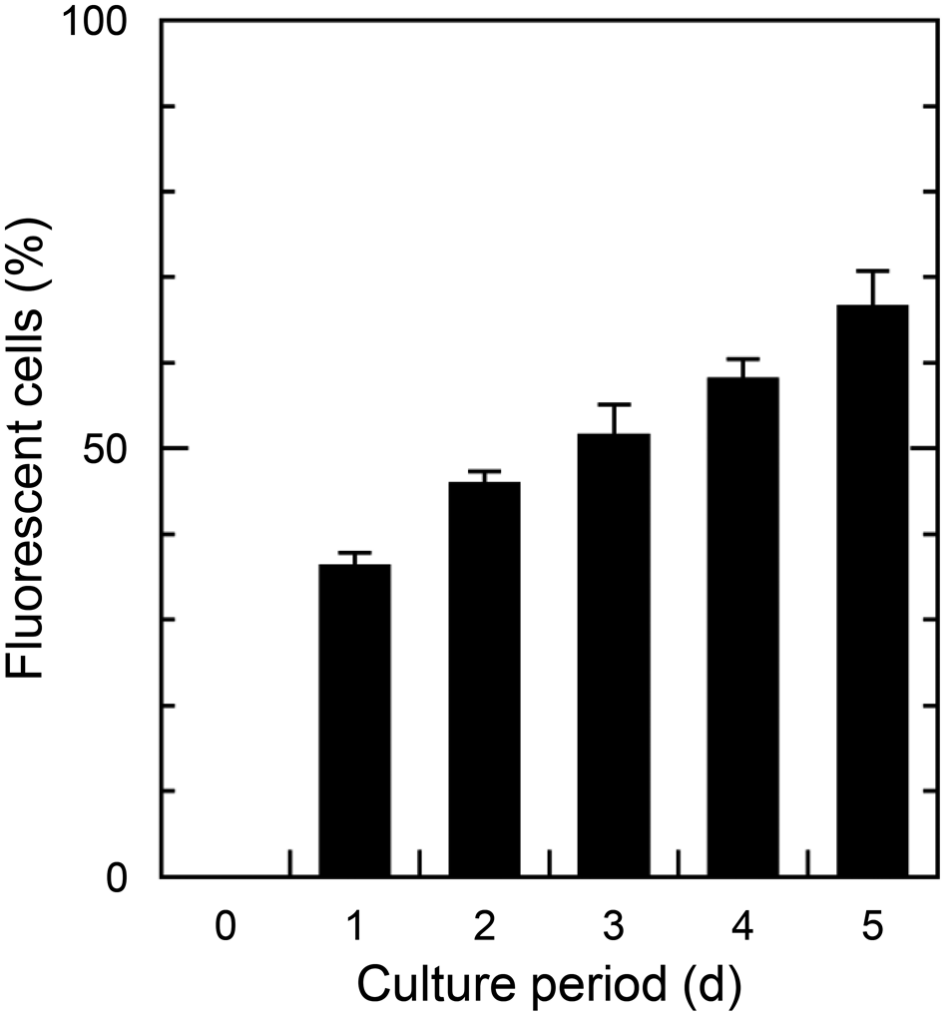
Incorporation of silicon detected by PDMPO fluorescence during the cell wall regeneration process of *Triparma laevis* NIES-2565. Cells that had lost all plates when cultured in 1 µM-silicate medium were transferred to 100 µM-silicate medium containing PDMPO at 0 d. Results are the mean ± SD of triplicate cultures.

**Figure 8:**
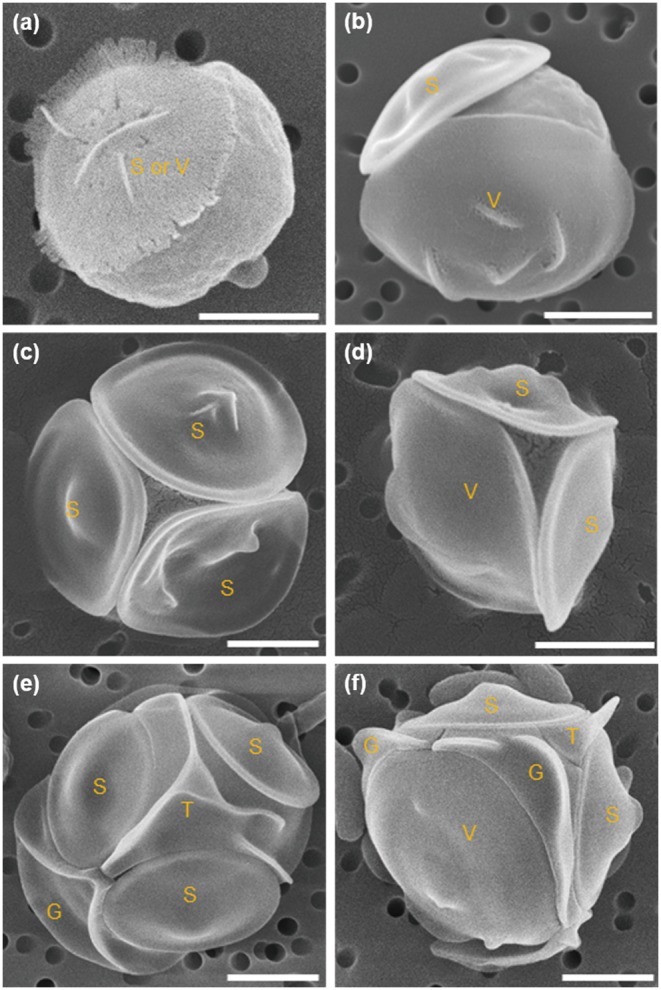
Various morphologies of siliceous plates observed during the cell wall regeneration process of *Triparma laevis* NIES-2565. Cells lost all plates, which cultured in 1 µM-silicate medium were transferred to 100 µM-silicate medium, and SEM observations were performed with cells collected within 24 h incubation. (a) Cell with immature shield (S) or ventral (V) plate that has fimbriate edge. (b) Cell with shield and ventral plates. (c) Cell with almost mature shield plates but without a triradiate plate. (d) Cell with almost mature ventral and shield plates but without a girdle plate. (e), (f) Cells with all plates regenerated. G: girdle plate; T: triradiate plate. Bars indicate 1 µm.

Siliceous plates lost when growing in low silicon concentrations could be regenerated by adding sufficient silicate. The process of plate regeneration was observed using PDMPO incorporation after naked type cells grown in 1 µM-silicate medium were transferred into 1 or 100 µM-silicate medium containing PDMPO. The naked type cells cultured in 1 µM-silicate medium did not incorporate PDMPO (Figure 6a, b). After 24 h incubation in 100 µM-silicate medium, about 36.3% cells incorporated PDMPO (Figures 6c, d, 7). Analysis of SEM images of cells after 24 h incubation in 100 µM-silicate medium revealed that the ratio of cells with regenerated plate(s) was almost the same as that estimated using PDMPO incorporation methods (34%, data not shown). The number of cells that incorporated PDMPO gradually increased when incubation in 100 µM-silicate medium was continued (Figure 7). Unfortunately, we could not detect the increase of PDMPO-fluorescent cells after 5 d incubation, because PDMPO in the medium was consumed and/or disrupted. SEM images revealed that more than 80% of cells were covered with mature plates after 12 d incubation (data not shown). Cells observed during the early stage (within 24 h incubation) of the wall regeneration process are shown in Figure 8; these were cells with one immature shield or ventral plate which had a fimbriate edge (Figure 8a), with almost mature shield and/or ventral plates (Figure 8b–d) and with mature all plates (Figure 8e–f). Here again, there was no cell with only girdle and/or triradiate plate(s); shield and ventral plates regeneration always preceded girdle and triradiate plate formation.

## Discussion

This is the first study to show the effect of silicon-limitation on physiological and morphological properties of a Parmales species, *Triparma laevis* NIES-2565. Among the artificial media we have tested, *T. laevis* grew well only in Aquil medium without addition of Tris-HCl. Although several artificial sea water medium described by Provasoli (1957) contain Tris-HCl to stabilize the pH of media at around 8.0 (same as sea water) [24], using a medium without Tris-HCl is preferable for *T. laevis* cells.Tris-HCl may inhibit their growth, as also reported in other marine pelagic organisms [20]. The pH of the medium was successfully maintained at around 8.0 throughout the culture period without Tris-HCl. The medium Aquil was developed for the study of trace metal requirements of marine phytoplankton and successfully used in coastal and oceanic species [20], [25]. Using this medium and polycarbonate culture vessels enabled us to study the effect of very low silicate concentrations.

*T. laevis* normally had a complete set of 8 plates, regardless of growth rate when sufficient quantities of silicon were available (100 µM-silicate medium). This observation suggests that when high quantities of silicon is available, plate formation and the cell division are coupled. Cells lost girdle and triradiate plates (non-round shape plates) first when silicate concentration in the medium was reduced. During the plate regenerating process, formation of shield and ventral plates always preceded girdle and triradiate plate regeneration. Results suggest that plate formation started with the shield and/or ventral plate followed by girdle and/or triradiate plates at a certain point in the cell cycle. Under silicon-limitation, cell division continued but with incomplete plate formation and loss of girdle and triradiate plates first. Cells lost all their plates and became ‘naked type’ when silicate concentration became too low even for forming shield and ventral plates. Although 66% cells in medium containing 6.8 µM silicate had one or more plate(s), 84% cells had no plates in medium containing 1.1 µM silicate. These results suggested that *T. laevis* was not able to form any siliceous plate in somewhere lower than 6.8 µM of silicate concentration.

Although the cellular silicon contents altered depending on ambient silicon concentrations, *T. laevis* continuously grew under silicon-limiting conditions with the same growth rate as that under silicon-sufficient conditions. Results indicate that silicon-limitation does not affect the growth of *T. laevis.* In most diatoms, silicon is essential for growth; cells stop dividing at the G-phase of cell cycle when sufficient quantities of silicon for frustule formation were not available [11], [12], [13], [14], [15]. Non-dividing cells with incomplete frustules are seldom observed during vegetative growth of diatoms [26], [27]. The only exception is the sperm of centric diatom, which does not have a siliceous wall, but there is no correlation between ambient silicon concentrations and sperm formation [28]. The polymorphic diatom *Phaeodactylum tricornutum* [29], [30] shows three different morphotypes, oval, fusiform and triradiate. Fusiform and triradiate cells grew without valves as cells of these stages had only girdle bands. However, interconversion between valve-forming (oval) and valve-less (fusiform and triradiate) stages of *P. tricornutum* seems not to be regulated by silicon concentrations [29]. There are few studies on the effects of silicon-limitation on siliceous-walled algae in heterokontophyta. *Synura petersenii* (Synurophyceae) grown in continuous cultures system kept growing even when cellular silicon concentrations became reduced to more than one-tenth of the normal level [16]. Under such silica limitation, scale deposition was completely suppressed and cells appeared to be scale-free. This depression of scale deposition was reversible, similar to *T. laevis*. Scale-free cells regenerated new scale layers upon silicate addition.

During the early stage of cell wall regeneration in *T. laevis*, newly formed shield and ventral plates were in contact with each other at their edge, and girdle and triradiate plates appeared as if they had pushed the shield and ventral plate aside. Girdle and triradiate plates may play a role to interlock among ventral and shield plates. More precise analysis using TEM during the process of *de novo* plate formation is necessary to prove this possibility. Interestingly, a regenerated plate with the fimbriate edge of *T. laevis* resembles the scales of centric diatom auxospores rather than the frustules of their vegetative cells [31].

The differences of silicon-limitation effect on cell division and wall formation between diatom and *T. laevis* suggest that the properties mentioned above, i.e., strong coupling between siliceous wall (frustule) formation and cell cycle was independently acquired in diatoms. This coupling may provide an advantages to diatom, because siliceous wall is thought to have several functions that protect cells from predator attack [32], [33], control of the sinking rate [34], absorb UV-irradiance [35] and/or facilitates the activity of extracellular carbonic anhydrase [36].

Silicon concentrations in the oceans that Parmales inhabit often decrease to less than 10 µM. In such waters, some Parmales cells may be produced without siliceous plates. As the estimation of biomass and distribution of Parmales has been based on their unique plate morphology using SEM images [37], [38], [39], it is likely that their reported distribution and biomass are under-estimated. Morphologically-independent methods, such as quantitative-PCR and fluorescence *in situ* hybridization are necessary to give better estimates of the actual biomass of Parmales.

## Supporting Information

Figure S1 **Growth and changes of silicate concentration in the medium during batch culture of** ***Triparma laevis*** **NIES-2565.** Cells of exponential growth phase in 10 µM-silicate medium were inoculated to the same medium at 0 d. Open circles and closed squares are for cell density and silicate concentration, respectively. Results are the mean ± SD of triplicate cultures. Error bar was omitted when ± SD was too small to be shown.
(TIF)
