# Supplementary figures and images for: Effects of Silicon-Limitation on Growth and Morphology of Triparma laevis NIES-2565 (Parmales, Heterokontophyta)
Source: PLoS One. 2014 Jul 23;9(7):e103289. doi: 10.1371/journal.pone.0103289 (PMC4108440; doi:10.1371/journal.pone.0103289)

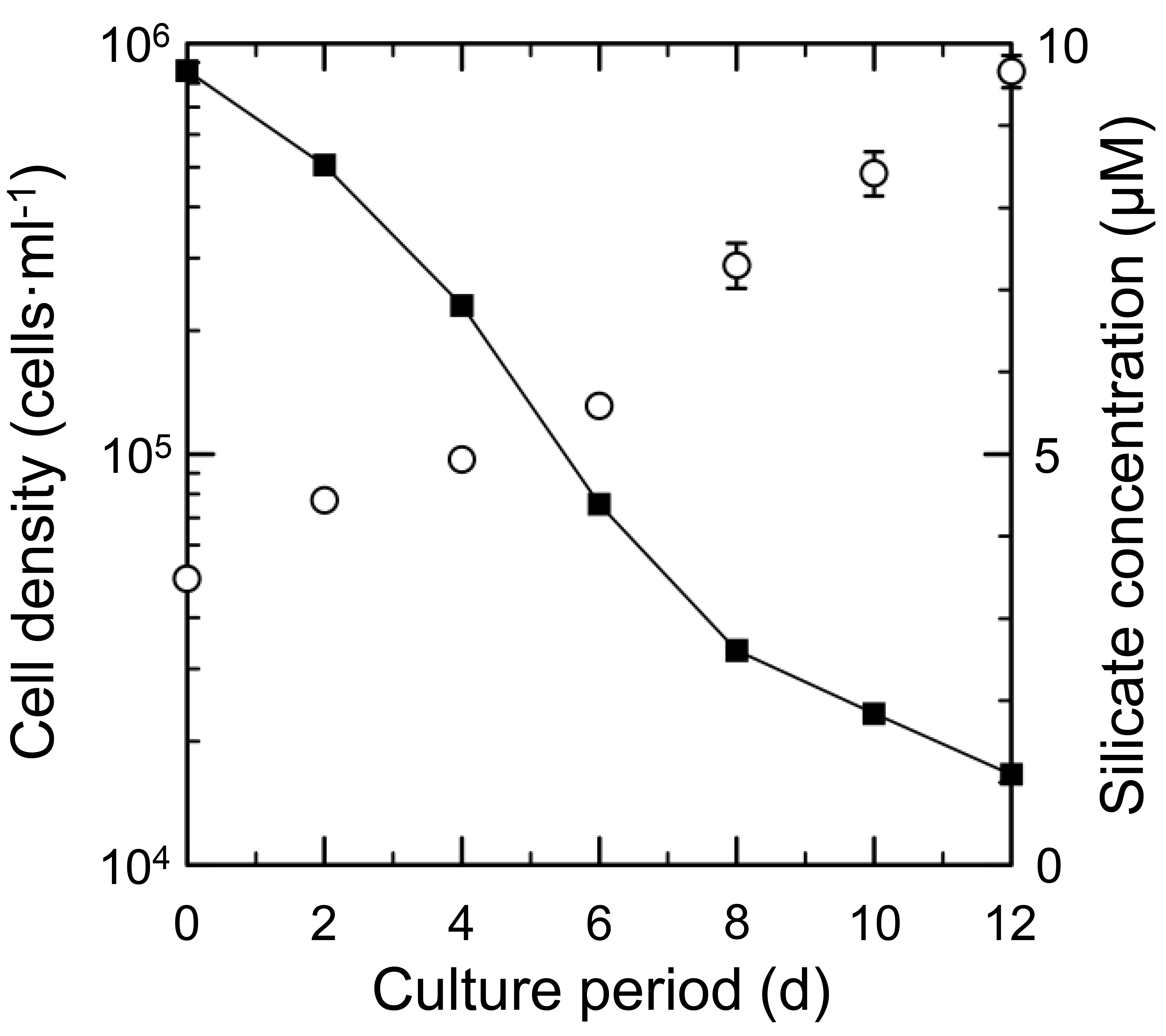

Supplement: Figure S1 — Growth and changes of silicate concentration in the medium during batch culture of Triparma laevis NIES-2565. Cells of exponential growth phase in 10 µM-silicate medium were inoculated to the same medium at 0 d. Open circles and closed squares are for cell density and silicate concentration, respectively. Results are the mean ± SD of triplicate cultures. Error bar was omitted when ± SD was too small to be shown. (TIF) [file pone.0103289.s001.tif]
